# Lesions of retrosplenial cortex spare immediate-early gene activity in related limbic regions in the rat

**DOI:** 10.1177/2398212818811235

**Published:** 2018-11-13

**Authors:** Anna L Powell, Emma Hindley, Andrew JD Nelson, Moira Davies, Eman Amin, John P Aggleton, Seralynne D Vann

**Affiliations:** School of Psychology, Cardiff University, Cardiff, UK

**Keywords:** c-*fos*, cingulate, hippocampus, subiculum, *zif268*

## Abstract

The retrosplenial cortex forms part of a network of cortical and subcortical structures that have particular importance for spatial learning and navigation in rodents. This study examined how retrosplenial lesions affect activity in this network by visualising the expression of the immediate-early genes c-*fos* and *zif268* after exposure to a novel location. Groups of rats with extensive cytotoxic lesions (areas 29 and 30) and rats with lesions largely confined to area 30 (dysgranular cortex) were compared with their respective control animals for levels of c-*fos* expression measured by immunohistochemistry. These cortical lesions had very limited effects on distal c-*fos* activity. Evidence of a restricted reduction in *c-fos* activity was seen in the septal dentate gyrus (superior blade) but not in other hippocampal and parahippocampal subareas, nor in the anterior cingulate and prelimbic cortices. Related studies examined *zif268* activity in those cases with combined area 29 and 30 lesions. The only clear evidence for reduced *zif268* activity following retrosplenial cell loss came from the septal CA3 area. The confined impact of retrosplenial tissue loss is notable as, by the same immediate-early gene measures, retrosplenial cortex is itself highly sensitive to damage in related limbic areas, showing a marked c-*fos* and *zif268* hypoactivity across all of its subareas. This asymmetry in covert pathology may help to explain the apparent disparity between the severity of learning deficits after retrosplenial cortex lesions and after lesions in either the hippocampus or the anterior thalamic nuclei.

## Introduction

Retrosplenial cortex (areas 29 and 30) provides an interface linking hippocampal, parahippocampal, parietal, frontal and anterior thalamic regions (Bubb et al., 2017; Jones and Witter, 2007; Kobayashi and Amaral, 2007; Morris et al., 1999; Shibata et al., 2004; van Groen and Wyss, 2003). This same cortical region is often placed within a sequence of inter-related limbic structures that support shared cognitive functions (Bubb et al., 2017; Delay and Brion, 1969; Papez, 1937). The properties of this network and its multiple interactions remain, therefore, of considerable interest (Bubb et al., 2017; Dalgleish, 2004; Vann et al., 2009). This study examined how structures within this network might depend on the integrity of retrosplenial cortex, using immediate-early gene (IEG) expression as an index of neural activity (Guzowski et al., 2001; Herdegen and Leah, 1998; Tischmeyer and Grimm, 1999).

Functions associated with retrosplenial cortex include episodic memory, spatial memory and navigation, as well as select aspects of cognitive control (Cooper et al., 2001; Hindley et al., 2014b; Keene and Bucci, 2008; Maguire, 2001; Miller et al., 2014; Nelson et al., 2014; Vann et al., 2003, 2009). In addition, retrosplenial cortex is one of the first brain regions to show metabolic hypoactivity and volume changes in Mild Cognitive Impairment and Alzheimer’s disease (Aggleton et al., 2016; Buckner et al., 2005; Chetelat et al., 2005; Minoshima et al., 1997; Nestor et al., 2003a, 2003b; Pengas et al., 2010; Scahill et al., 2002). These factors highlight the need to understand more about how retrosplenial cortex pathology might affect other brain sites.

This study asked whether selective cytotoxic retrosplenial lesions in rats induce IEG activity changes across related brain areas. Taking advantage of the specificity possible with rats, this study included surgeries that targeted just the dysgranular retrosplenial cortex (area 30), as well as more extensive lesions of both granular and dysgranular retrosplenial cortex (areas 29 and 30). While area 29 has more interconnections with hippocampal and parahippocampal areas, area 30 has relatively more connections with cortical visual areas and has more inputs to the anteromedial thalamic nucleus (Shibata, 1998; Sugar et al., 2011; van Groen and Wyss, 1990, 1992, 2003).

The consequences of retrosplenial cortex damage were mapped by measuring expression of the IEGs c-*fos* and *zif268* (also known as EGR-1 and NGFI-A) in both cortical and subcortical sites. To induce IEG expression, rats ran up and down pre-selected arms of a radial maze (RAM) in a novel room. Variants of this procedure reliably induce IEG activation in hippocampal-related areas, including retrosplenial cortex (Frizzati et al., 2016; Pothuizen et al., 2009; Vann, 2013; Vann and Albasser, 2009). These two IEGs (c-*fos* and *zif268*) were selected as they provide indirect markers of neuronal activity that have been repeatedly linked to learning, including different aspects of spatial memory (Cowansage et al., 2014; Flavell and Greenberg, 2008; Gallo et al., 2018; Guzowski et al., 2001; Liu et al., 2012; Mendez et al., 2015; Tischmeyer and Grimm, 1999; Xu and Sudhof, 2013).

Levels of retrosplenial c-*fos* and *zif268* expression are highly dependent on the integrity of other limbic sites. These sites include the anterior thalamic nuclei, Gudden’s ventral tegmental nucleus, the mammillothalamic tract, the lateral mammillary nuclei, and the hippocampus (Albasser et al., 2007; Dalrymple-Alford et al., 2015; Dumont et al., 2012; Frizzati et al., 2016; Jenkins et al., 2002a, 2002b; Poirier and Aggleton, 2009; Vann, 2013; Vann, 2018; Vann and Albasser, 2009). These findings raise the question of whether this disconnection sensitivity reflects a general property of these interlinked areas or whether it is a special feature of retrosplenial cortex.

## Material and methods

The study involved two different cohorts. The first cohort contained rats with area 30 lesions (RSCdysg), while the rats in the second cohort had more extensive lesions that targeted both areas 29 and 30 (RSCcomb). The methods for both lesion groups, and their controls, were very similar. Nevertheless, one difference is that additional brain areas were examined for *c-fos* activity in the RSCcomb cohort in response to the many null results found in the RSCdysg cohort. In addition, *zif268* expression was only measured in the RSCcomb cohort.

### Subjects

The subjects were male Lister Hooded rats (Harlan UK Ltd, Bicester, UK). At the time of surgery, the RSCdysg rats (Cohort 1) weighed between 294 and 314 g, while the RSCcomb rats (Cohort 2) weighed between 309 and 356 g. The rats in Cohort 1 were housed in pairs, while rats in Cohort 2 were kept in groups of four. All rats were housed in a temperature-controlled room in which lighting was on a 12-h light/dark cycle (light from 08:00 to 20:00), with testing during the light period.

Prior to the current experiment, both cohorts experienced various learning tasks as part of separate behavioural research. The RSCdysg rats were tested on cross-modal recognition and object recognition (Hindley et al., 2014a), as well as spatial discrimination learning (Hindley et al., 2014b). Meanwhile, the RSCcomb rats were tested on object recognition and recency (Powell et al., 2017b) and operant visual and response discriminations (Powell et al., 2017a) and trained on a cost-benefit task (8 months post surgery; Powell et al., 2017a). For both cohorts, the interval between the last behavioural task mentioned above and the behaviour for the IEG study was at least 2 weeks. Water was available *ad libitum* in the home cages, in which the rats were also provided with cardboard tubes and wooden chew sticks. For both experiments, rats were food restricted to approximately 85% of their free-feeding body weight and were maintained at this level or above. All experiments were in accordance with the UK Animals (Scientific Procedures) Act (1986) and associated guidelines, as well as EU directive 2010/63/EU. The study was also approved by local ethical review committees at Cardiff University.

### Surgical procedures

Cohort 1 rats received either bilateral excitotoxic lesions involving area 30 (RSCdysg, *n* = 14) or a surgical control procedure (Sham1, *n* = 10). Animals in Cohort 2 received either bilateral excitotoxic lesions of areas 29 and 30 (RSCcomb, *n* = 16) or were surgical controls (Sham2, *n* = 16).

Both cohorts were deeply anaesthetised with an intra-peritoneal (i.p.) injection of sodium pentobarbital ((RSCdysg: 60 mg/kg pentobarbital sodium salt (Sigma-Aldrich, Pool, UK); RSCcomb: 1 mL/kg, i.p. injection of 6% sodium pentobarbital solution (Ceva Animal Health, Libourne, France)). At the start of surgery, all animals received a subcutaneous injection of 0.06 mL Metacam (Boehringer Ingelheim, Alkmaar, NL, USA) to reduce post-operative pain. Each rat was placed in a stereotaxic frame (David Kopf Instruments, Tujunga, CA, USA) with the nose bar set at +5.0. The skull was exposed, followed by a bilateral craniotomy above the midline, which extended from bregma to lambda.

Cytotoxic lesions were made by injecting 0.09M *N*-methyl-d-aspartate (NMDA; Sigma, Poole, UK) dissolved in phosphate-buffered saline (PBS; pH 7.2) into seven sites per hemisphere, each at a rate of 0.05 µL/min using a 1 µL Hamilton syringe (gauge 25 s; Bonaduz, Switzerland). The stereotaxic coordinates are given relative to bregma in the anterior–posterior (AP) axis and relative to the central sinus in the lateral–medial (LM) axis. Dorsal–ventral (DV) coordinates are relative to the surface of the cortex, using the eye of the needle as a reference point.

Stereotaxic coordinates for the RSCdysg lesions were as follows: AP −1.6, LM ±0.4, DV −1.0; AP −2.8, LM ±0.5, DV −1.1; AP −4.0, LM ±0.5, DV −1.1; AP −5.3, LM ±0.5, DV −2.4; AP −5.3, LM ±0.9, DV −1.4; AP −6.6, LM ±0.9, DV −1.8; AP −7.5, LM ±1.0, DV −1.1. At each site, 0.25 µL NMDA was injected, apart from the most caudal pair of injections, where the injections were 0.1 µL NMDA. The RSCcomb coordinates were as follows: −1.8 (AP), ±0.5 (ML), −1.0 (DV); −2.8 (AP), ±0.5 (ML), −1.1 (DV); −4.0 (AP), ±0.5 (ML), −1 (DV); −5.3 (AP), ±0.5 (ML), −2.5 (DV); −5.3 (AP), ±0.9 (ML), −1.4 (DV); −6.6 (AP), ±0.9 (ML), −1.8 (DV). A volume of 0.25 μL of NMDA was injected in the most anterior three pairs of sites, while 0.27 μL was injected in the remaining sites. All animals in the RSCcomb cohort also received an injection of atropine (0.06 mL of a 600 μg/mL solution; Martindale Pharma, Brentwood, UK).

After each NMDA infusion, the needle remained in place for 5 min before being slowly withdrawn. Following surgery, the scalp was sutured and a subcutaneous injection of 5 mL glucose-saline given. Lidocaine (Xylocaine; Pfizer, Sandwich, UK) and antibiotic powder (Dalacin C; B.Braun, Melsungen, Germany) were applied topically to the wound. Sham surgeries consisted of the same procedures (i.e. anaesthesia, placement in stereotaxic apparatus, craniotomy and subsequent suturing), except that the needle was not lowered in the cortex and so injections were not made. Post-operative care was identical for all groups. Animals recovered well following all surgeries.

### Behavioural procedures

#### Apparatus

Behavioural testing was conducted in an eight-arm RAM. The maze consisted of eight equally spaced radial arms (87 cm long, 10 cm wide) and an octagonal central platform (34 cm diameter). The walls of the arms were made from clear Perspex (24 cm high), while the base of the central platform and the arms were made of wood. There was a food well 2 cm in diameter and 0.5 cm deep at the end of each arm. Access in and out of the central platform was controlled by 12 cm high clear Perspex sliding doors at the start of each arm, controlled by the experimenter.

Two identical RAMs were placed in two different rooms. The two rooms were markedly different (i.e. size, shape, lighting) and each had salient visual cues, such as geometric shapes and high contrast stimuli, on the walls. All animals were first trained in the same room (258 × 343 cm^2^) and then tested on the final day in a novel room (300 × 300 cm^2^). Animals were always transported in an individual, opaque travel box with aluminium top, base and sides (10 × 10 × 26 cm^3^).

#### Behavioural training

Cohort 1 began training 12 months post surgery, while Cohort 2 began training 9 months post surgery. Before starting behavioural training, all rats were habituated to a dark room in two 30 min sessions on consecutive days. For this period, the rats were placed individually in a cage, similar to their home cage, in a dark, quiet room. Each animal was allocated the same cage for all stages of the experiment.

Next, training in a RAM commenced. All animals were trained to run down a predetermined sequence of arms, with only one arm choice available for each run, that is, there was no working memory demand. Three training days were given. This task was selected as the retrosplenial lesion groups would likely behave differently from the control groups if given a working memory task (Vann and Aggleton, 2002, 2004), making it difficult to interpret subsequent IEG expression. Immediately before each RAM session, animals were placed in the dark for 30 min (as described above) and then completed one forced-entry RAM session per day, which lasted for a maximum of 20 min. Typically, this session consisted of four ‘trials’ (occasionally five). To complete a ‘trial’, the rat ran down the eight arms of the maze, in a pre-selected random order, to retrieve a single sucrose pellet (45 mg; Noyes Purified Rodent Diet, Lancaster, NH, USA), previously placed in the food well at the end of each arm. Arm entry was controlled by the experimenter, using a pulley system, such that only one arm was open at any given time. After 3 days of RAM training, each involving different arm sequences, the final session occurred. This session was the same as those before, but it took place in the novel room. The final training day and test day were always on consecutive days.

For all sessions, each animal was placed in the centre of the RAM for 10 s before the first arm was opened. Once the animal had entered the arm, the sliding door was lowered until the sucrose pellet had been retrieved, after which the door was opened and the animal made its way to the centre of the maze. After a further 10 s delay, the next door was opened. This procedure continued until all eight arms had been visited. At the end of each trial, the animal was returned to the travel box, while the arms were rebaited (~2 min). After 20 min, the session was terminated and the animal was returned to the dark room for 90 min. On the final test day, after 90 min in the dark, the animal was anaesthetised and perfused. This interval (90 min) overlaps with peak c-Fos and Zif268 protein levels after initial activation (Richardson et al., 1992; Sharp et al., 1993; Zangenehpour and Chaudhuri, 2002).

### Histological procedures

On the final test day, 90 min after behavioural testing, all animals were deeply anaesthetised using sodium pentobarbital (60 mg/kg, i.p.; Euthatal; Merial Animal Health, Harlow, UK), before being transcardially perfused with 0.1 M PBS followed by 4% paraformaldehyde in 0.1 M PBS (PFA). The brains were post-fixed in PFA for 4 h before being transferred to a 25% sucrose solution and left overnight at room temperature, with gentle agitation. Sections were cut at 40 µm on a freezing microtome. A one-in-four series was mounted directly onto gelatine-coated slides and stained using cresyl violet to enable visualisation of the specific brain regions and lesion site. A further one-in-four series was collected in PBS and processed for c-Fos protein immunohistochemistry.

### Fos immunohistochemistry

Before processing, the lesions were assessed to determine whether they were acceptable. To minimise the variance in cell staining, all of the sections for key regions in Cohort 1 were stained simultaneously (lesion and sham control cases). For this reason, all septal hippocampal sections were processed together. Likewise, all medial prefrontal sections were treated together. Although the caudal anterior cingulate and visual cortices were processed in different batches, an equal number of lesion and control cases were processed together in each batch.

The procedure was modified for Cohort 2 as the larger group numbers made it impractical. For this reason, all tissues from an individual animal were processed simultaneously. However, each acceptable lesion case was matched to a sham control animal with comparable behavioural performance in the RAM, that is, the comparable number of trials completed in 20 min. These pairs of animals received their immunohistochemical staining at the same time in the same solutions, so producing matched sets of tissue (13 in total).

The subsequent histological procedures were the same for the two cohorts, except that different primary Fos antibodies were used for each cohort (RSCdysg: Synaptic Systems, Goettingen, Germany; RSCcomb: Merck Millipore, Hertfordshire, UK) with incubation times optimised to the specific antibody (see details below). After three washes in PBS (each wash, and all subsequent washes, for 10 min), sections were incubated in a 0.3% hydrogen peroxide /10% methanol PBS solution for 10 min. The sections were then washed four times in alternating PSB/PBS-Triton X 0.3% (PBS-TX) solutions. To prevent non-specific antibody binding, the sections were then incubated for 1 h in 10% normal goat serum (NGS) in PBS-TX. After a further PBS-TX wash, the sections were incubated in Fos rabbit polyclonal primary antibody at 1:10,000 dilution in 3% NGS and PBS-TX for 48 h at 4°C (RSCdysg) or 24 h at room temperature (RSCcomb).

Following a further five alternating PBS-TX/PBS washes, sections were incubated in the secondary antibody, biotinylated goat anti-rabbit (1:200 in PBS-TX; Vector Laboratories, Burlingame, CA, USA) and 3% NSG for 2 h. After a further five alternating PBS-TX/PBS washes, they were processed with avidin-biotinylated horseradish peroxidase complex in PBS-TX (Elite ABC Kit; Vector Laboratories) for 1 h at room temperature. Finally, sections were washed once in PBS-TX and twice in PBS, followed by two washes in 0.05 M Tris non-saline (TNS) buffer. The reaction was then visualised using diaminobenzidine (DAB Substrate Kit; Vector Laboratories) and stopped by washing in cold TNS. Processed sections were mounted on gelatine-coated slides, dehydrated through a graded series of alcohols (70%, 90% and 100%) and cover-slipped.

#### Fos-positive cell counts

Estimates of the number of Fos-positive cells in each region of interest were made using an automated cell counting procedure. Wherever possible, cell counting occurred without knowledge of the group assignments. (This blind procedure was not always possible for those sections that also contained lesioned areas.) Images were viewed on a Leica DMRB microscope and photographed using an Olympus DP70 camera. The programme cellSens (Olympus) counted the number of cells stained above a threshold of greyscale intensity that was above background level.

These counting procedures are not stereological and therefore do not provide absolute cell numbers, but rather provide a relative measure of numbers of IEG-positive cells, with various caveats (Coggeshall and Lekan, 1996). For all brain areas analysed, counts were taken from either three or four sections per hemisphere, depending on the brain area. The number of sections from a given area was the same for all animals. The cells counts for a given area were then averaged to produce a mean count.

#### Regions of interest

**Figure 1.**
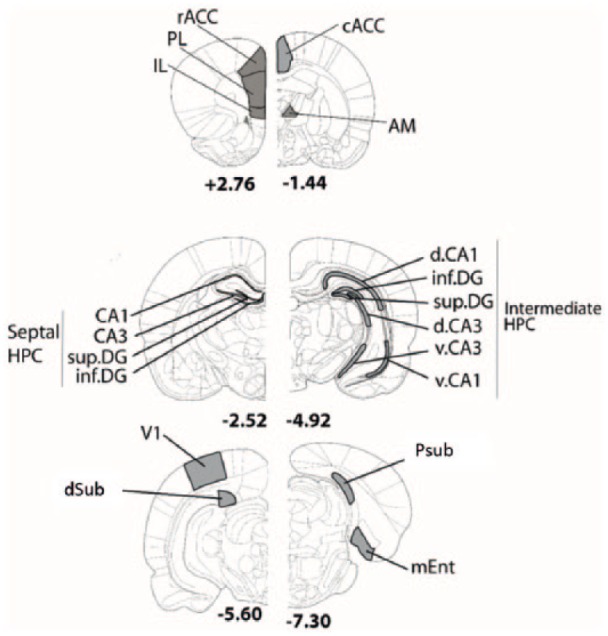
Schematic representation showing the regions of interest for cell counts. cACC: caudal anterior cingulate; rACC: rostral anterior cingulate; AM: anteromedial thalamic nucleus; dCA1: dorsal CA1; vCA1: ventral CA1; d.CA3: dorsal CA3; v.CA3: ventral CA3; inf.DG: inferior dentate gyrus; sup.DG: superficial dentate gyrus; mEnt: medial entorhinal cortex; IL: infralimbic cortex; PL: prelimbic cortex; Psub: post subiculum; dSub: dorsal subiculum; V1: visual cortex. The numbers refer to distance from bregma (Paxinos and Watson, 2005).

Cytoarchitectonic subfields were identified from coronal sections using the nomenclature of Paxinos and Watson (1997). Outlines of all the regions sampled are depicted in Figure 1. These brain regions were chosen on the basis of their anatomical or functional connectivity with retrosplenial cortex. Many of the same regions normally show c-*fos* and *zif268* activity changes after running in a radial-arm maze (Pothuizen et al., 2009; Vann, 2013; Vann and Albasser, 2009; Vann et al., 2000a, 2000b). Within the hippocampus, attention focussed on its dorsal (septal) regions as this part of the structure is reciprocally connected with retrosplenial cortex (Wyss and van Groen, 1992).

In Cohort 1 (area 30 lesions) and Cohort 2 (area 29 + 30 lesions), counts of hippocampal Fos-positive cells came from the septal CA1 and CA3 subfields and the adjacent dentate gyrus (DG; Figure 1). Within the DG, separate counts were taken from the superior and inferior blades (sup.DG and inf.DG; Figure 1). Other counts were taken from prelimbic (PL) cortex, infralimbic (IL) cortex, rostral and caudal anterior cingulate cortex (rACC/cACC respectively) and primary visual cortex (V1).

In Cohort 2, additional cell counts took place in other hippocampal areas, along with parts of the parahippocampal cortex. Consequently, additional Fos-positive cell counts were made in the intermediate hippocampus (see Bast, 2007). These counts in intermediate CA1 and CA3 were subdivided into dorsal and ventral regions (dCA1 and vCA1; dCA3 and vCA3, respectively, see Figure 1). Further Fos-positive cell counts were made in related cortices: the dorsal subiculum (dSub), post subiculum (Psub) and medial entorhinal cortex (mEnt). Finally, Fos counts in Cohort 2 were made in the anteromedial thalamic nucleus (AM; see Figure 1), but not in the anteroventral thalamic nucleus, where levels of c-*fos* expression are often very low and where retrosplenial cortex lesions cause retrograde cell loss within the nucleus (Neave et al., 1994; Warburton et al., 1998).

### Zif268 immunohistochemistry

The same basic processing and cell counting procedures described above were repeated on a separate series of sections from Cohort 2 for Zif268 immunohistochemistry. For Zif268 staining, sections were washed three times in PBS before incubation in a 3% hydrogen peroxide blocking solution. After blocking, sections were washed a further three times in PBS, followed by four washes in PBS-TX. The sections were then incubated for 1 h in 3% NGS in PBS-TX before being incubated in a 1:3000 primary antibody solution (Egr1, sc-189; Santa Cruz Biotechnology) in 1% NS, PBS-TX, for 48 h at 4°C. After a further three washes in PBS, the sections were transferred to the secondary antibody, biotinylated goat anti-rabbit (1:200 in PBS-TX, 1% NGS; Vector Laboratories) for 2 h. Sections were then washed three more times in PBS before being incubated in avidin-biotinylated horseradish peroxidase complex in PBS-TX (Elite ABC Kit; Vector Laboratories) for 2 h. Finally, sections were washed three times in PBS-TX and then twice in 0.05 M TNS buffer. The reaction was visualised using DAB Substrate Kit (Vector Laboratories) and stopped by washing in cold TNS.

In response to the many null results from the c-Fos data, the Zif268 cell counts focussed on those regions most interconnected with retrosplenial cortex, including any that had shown evidence of a change in c-Fos levels. For this reason, counts were made in the septal and intermediate hippocampus (CA1, CA3 and DG), dorsal subiculum, post subiculum and anterior cingulate cortices (rostral and caudal). Anterior thalamic cell counts were not included as the number of Zif268-positive cells was too low.

### Data analysis

All analyses were conducted on raw cell counts (i.e. Fos- or Zif268-positive cells). To test for lesion effects, a series of mixed analyses of variance (ANOVAs) were calculated, with the between-subjects factor Group (RSCdysg vs Sham1 (Cohort 1) or RSCcomb vs Sham2 (Cohort 2)) and the within-subjects factor region(s) of interest (ROI). Mauchly’s test of sphericity helped to determine whether the group variances were similar. Each analysis was carried out for four regional groupings, with the groupings limiting type 1 errors. These groupings were as follows: (1) septal hippocampus (CA1, CA3 and DG), (2) intermediate hippocampus (CA1, CA3 and DG), (3) hippocampal and parahippocampal cortices (mEnt, Psub and dSub) and (4) other cortical regions (IL, PL, rACC, cACC and V1). The cell counts from the anteromedial thalamic nucleus, a nucleus that does not align with just one of the other groups, were analysed separately with an independent *t*-test. Where *F* and *t* values were >1, generalised eta (*ges*) values were reported as an estimate of effect size (Bakeman, 2005; Olejnik and Algina, 2003). In general, a small effect size is defined as *ge*s = 0.02, a medium effect size as *ges* = 0.13 and a large effect size as *ges* = 0.26 or more (Cohen, 1977; Olejnik and Algina, 2003). All statistical analyses were conducted in R (V 3.3.2) using the ‘aov_ez()’ function within the ‘afex’ package and specifying Type 3 ANOVA.

In both the septal and intermediate hippocampus, the DG was further subdivided into its superior and inferior blades (sup.DG and inf.DG). Similarly, in intermediate hippocampus, CA1 and CA3 were further subdivided into dorsal (dCA1 and dCA3) and ventral (vCA1 and vCA3) regions. Counts from these additional subdivisions were analysed in separate ANOVAs using the design described above. Any significant interactions from the ANOVAs were further examined using a simple effects analysis to compare counts between groups in specific subfields.

## Results

### Lesion extent and location

#### Dysgranular lesion cohort

**Figure 2.**
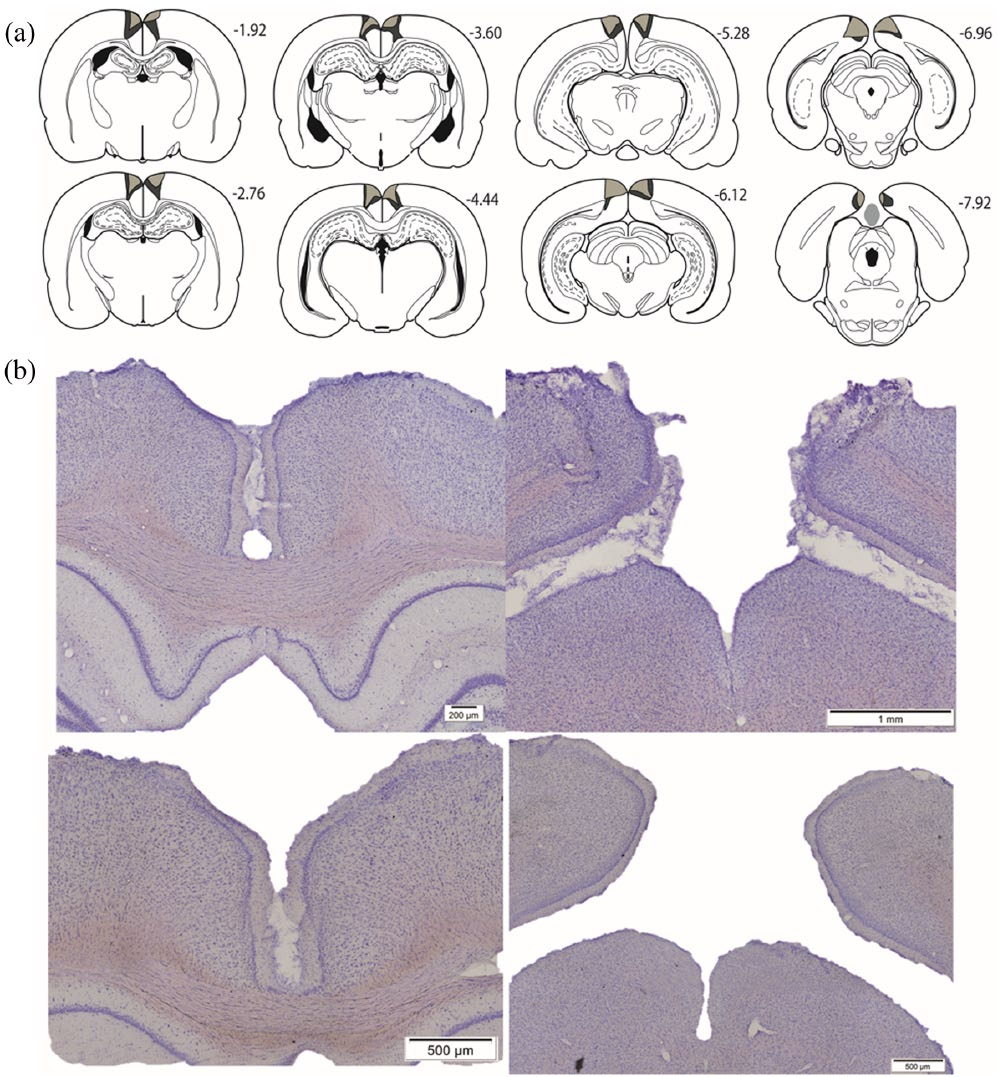
Extent and appearance of the dysgranular retrosplenial cortex lesions (Cohort 1). (a) The largest (pale grey plus dark grey) and smallest (pale grey) cortical lesions are depicted on a series of coronal sections. The numbers refer to the approximate distance in millimetre of each section caudal to bregma (Paxinos and Watson, 2005). (b) Photomicrographs from a representative lesion (top row) and a surgical sham case (bottom row).

Of the 14 cases, 6 rats in the RSCdysg group were excluded as the lesions were either only present in one hemisphere or because there was a high level of sparing of the dysgranular retrosplenial cortex. The final number of animals in the RSCdysg group was eight, with 10 in the corresponding sham group (Sham1). In the final RSCdysg group, no animal had damage to the hippocampus or the subiculum. Of the RSCdysg animals, 5 had a very limited amount of unilateral damage to the granular retrosplenial cortex (Rgb). The lesions did not extend into any other adjacent cortical areas. Following sectioning, tissue was lost from two lesion cases. For this reason, the final comparisons were between 6 RSCdysg cases and 6 paired Sham1 cases (Figure 2).

**Figure 3.**
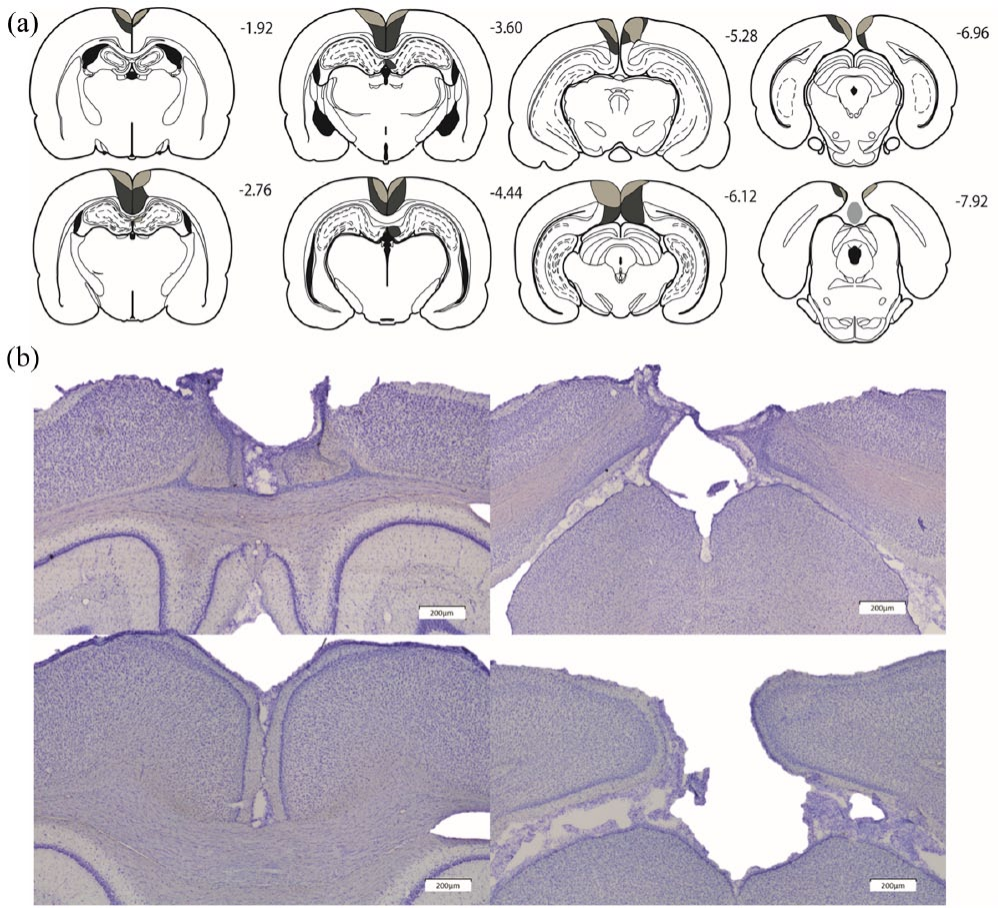
Extent and appearance of the combined (granular plus dysgranular) retrosplenial cortex lesions (Cohort 2). (a) The largest (pale grey plus dark grey) and smallest (pale grey) cortical lesions on a series of coronal sections. The numbers refer to the approximate distance in millimetres of each section caudal to bregma (Paxinos and Watson, 2005). (b) Photomicrographs from a representative lesion (top row) and a surgical sham case (bottom row).

Of the 16 RSCcomb rats, 2 animals were excluded due to bilateral damage in dorsal CA1, while a further case was excluded because it had almost complete sparing of granular retrosplenial cortex. Of the remaining 13 animals, 8 had considerable cell loss in both the granular and dysgranular cortices, both anterior and posterior to the splenium (Figure 3). In some cases, this cell loss was particularly evident in more superficial cell layers. Only the most caudal parts of area Rga were spared in these cases. In 2 of these cases, there was a small amount of sparing in granular retrosplenial cortex close to the anterior cingulate border, while in 1 case, the lesion encroached into cACC. In 1 case, there was noticeable unilateral sparing of granular retrosplenial cortex anterior to the splenium; 3 cases had minimal unilateral cell loss limited to the dorsal medial CA1 below the rostral retrosplenial cortex.

The five remaining cases had lesions primarily centred on dysgranular cortex. In these five cases, cell loss was also evident in the deeper layers of the granular cortex. In total, the data from 13 RSCcomb and 13 Sham2 animals were compared for all ROI groupings apart from the hippocampal and parahippocampal cortices group (mEnt, psub and dSub). It was not possible to obtain counts in the medial entorhinal cortex in one animal from the RSCcomb group. Therefore, this animal and its paired sham were removed from this one analysis.

### Behaviour

In Cohort 1, the RSCdysg group entered fewer arms during the 20 min final test session than the Sham1 group (mean arm entries: RSCdysg = 24; Sham1 = 27.8), but this difference was not significant (*t*(10) = 2.13, *p* = 0.06). In Cohort 2, the RSCcomb and Sham 2 rats entered very similar numbers of arms (mean arm entries: RSCcomb = 31; Sham2 = 31.5; *t* < 1).

### IEG expression

**Figure 4.**
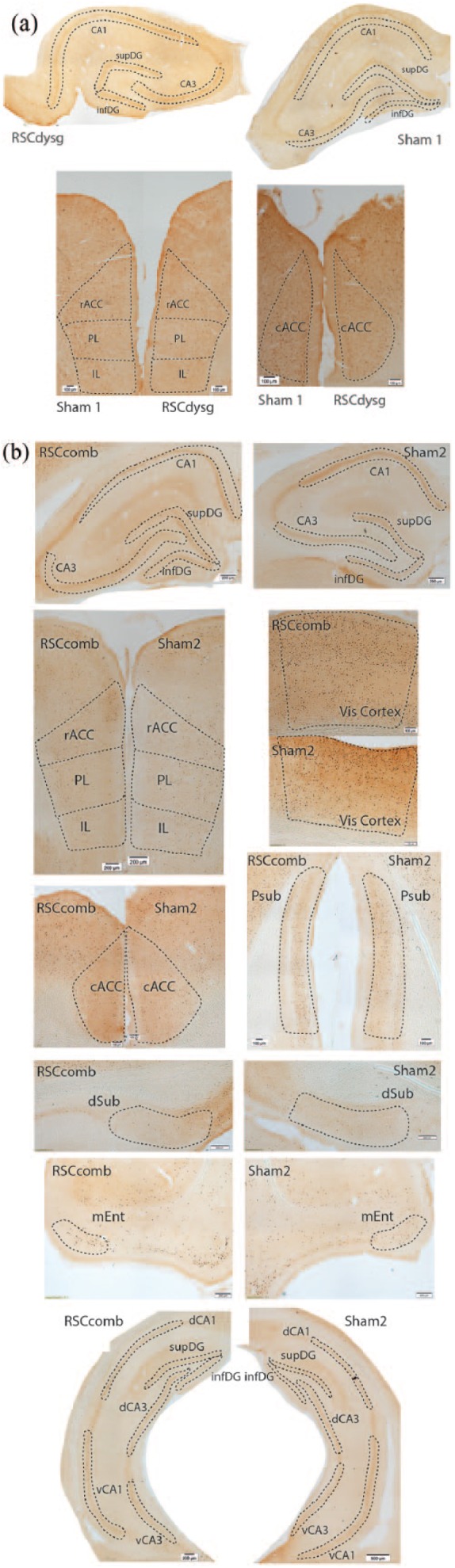
Coronal images of sections stained for Fos protein from Cohort 1 ((a) dysgranular retrosplenial lesion (RSCdysg) and sham control (Sham1)) and Cohort 2 ((b) combined granular and dysgranular retrosplenial lesion (RSCcomb) and sham control (Sham2)). cACC: caudal anterior cingulate; rACC: rostral anterior cingulate; AM: anteromedial thalamic nucleus; dCA1: dorsal CA1; vCA1: ventral CA1; dCA3: dorsal CA3; vCA3: ventral CA3; infDG: inferior dentate gyrus; supDG: superficial dentate gyrus; mEnt: medial entorhinal cortex; IL: infralimbic cortex; PL: prelimbic cortex; Psub: post subiculum; dSub: dorsal subiculum; V1: visual cortex.

Figure 4 shows the appearance of the c-Fos protein, visualised by immunohistochemistry, in the various regions of interest, with representative sections from Cohorts 1 (a) and 2 (b).

### Effects of dysgranular retrosplenial cortex lesions on distal c-Fos expression (groups RSCdysg and Sham1)

**Figure 5.**
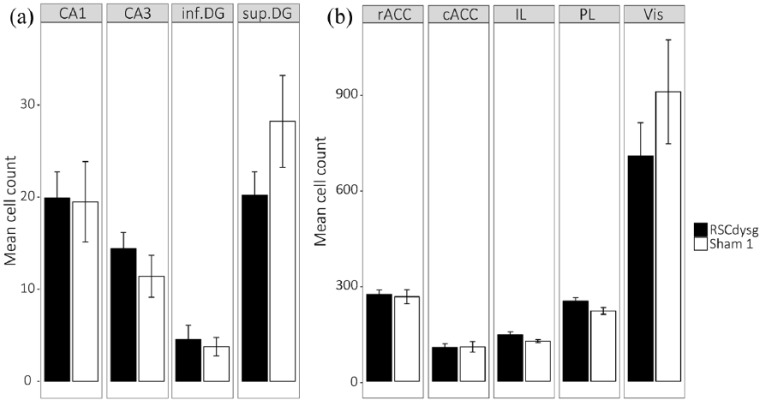
Mean Fos-positive cell counts after dysgranular retrosplenial cortex (RSCdysg) and sham control (Sham1) surgeries (Cohort 1). (a) Fos counts in septal hippocampal regions. (b) Fos counts in cortical regions. cACC: caudal anterior cingulate; rACC: rostral anterior cingulate; inf.DG: inferior dentate gyrus; sup.DG: superficial dentate gyrus; IL: infralimbic cortex; PL: prelimbic cortex; V1: visual cortex.

Starting in the septal hippocampus (CA1, CA3 and DG), there was no main effect of Group (*F* < 1) and no Group × ROI interaction (*F*(2, 20) = 1.73, *p* = 0.20; *ges* *=* 0.07; Figure 5(a)). Furthermore, when the counts in the superior and inferior blades of the DG were separated, there was again no main effect of Group (*F*(1, 10) = 1.28, *p* = 0.28; *ges* *=* 0.07) and no Group × ROI interaction (*F*(1, 10) = 2.68, *p* = 0.13; *ges* *=* 0.1; Figure 5(a)). For the cortical regions (prelimbic, infralimbic, anterior cingulate and visual cortices), there was again no main effect of Group (*F* < 1) nor Group × ROI interaction (*F*(4, 40) = 1.25, *p* = 0.31; *ges* *=* 0.09; Figure 5(b)).

### Effects of combined retrosplenial cortex lesions on distal c-Fos expression (groups RSCcomb and Sham2)

**Figure 6.**
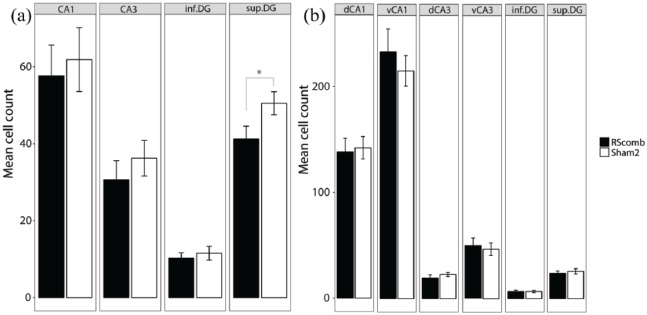
Mean Fos-positive cell counts after combined granular and dysgranular retrosplenial cortex (RSCcomb) and sham control (Sham2) surgeries (Cohort 2). (a) Fos counts in septal hippocampal regions. (b) Fos counts in intermediate hippocampal regions. dCA1: dorsal CA1; dCA3: dorsal CA3; vCA1: ventral CA1; vCA3: ventral CA3; inf.DG: inferior dentate gyrus; sup.DG: superficial dentate gyrus.

In the septal hippocampus (CA1, CA3 and DG), there was no main effect of Group (*F* < 1) and no Group × ROI interaction (*F* < 1; Figure 6(a)). However, when the counts in the superior and inferior blades of the DG were analysed separately, there was a significant Group × ROI interaction (*F*(1, 24) = 4.65, *p* = 0.04; *ges* *=* 0.05). Simple effects analysis revealed that this interaction was due to higher Fos-positive cell counts in the Sham2 group relative to the RSCcomb group, specifically in the superior blade of the DG (*F*(1, 24) = 4.29, *p* = 0.05; *ges* *=* 0.15; Figure 6(a)).

*P ≤ 0.05.

In the intermediate hippocampus, there was no main effect of Group (*F* < 1) and no Group × ROI interaction (*F* < 1; Figure 6(b)). When the counts in the superior and inferior DG were analysed separately, there was no main effect of Group (*F* < 1) and no Group × ROI interaction (*F* < 1; Figure 6(b)). Similarly, when CA1 and CA3 were split into dorsal and ventral subfields, there was no main effect of Group (*F* < 1) and no Group × ROI interaction (*F* < 1; Figure 6(b)).

**Figure 7.**
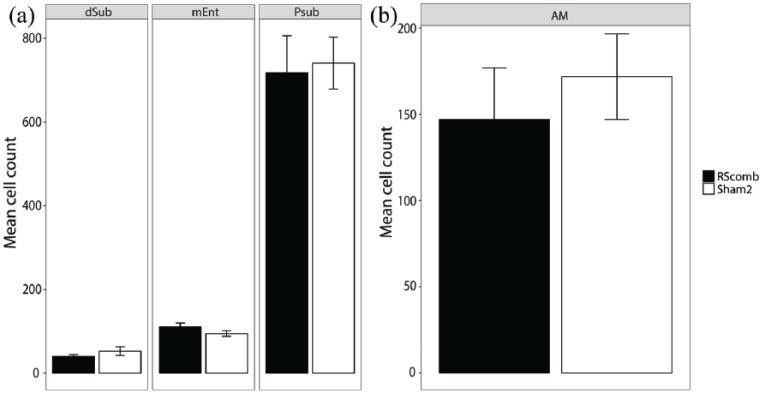
Mean Fos-positive cell counts after combined granular and dysgranular retrosplenial cortex (RSCcomb) and sham control (Sham2) surgeries (Cohort 2). (a) Additional hippocampal and parahippocampal sites. (b) Fos counts in anteromedial thalamic nucleus. AM: anteromedial thalamic nucleus; mEnt: medial entorhinal cortex; Psub: post subiculum; dSub: dorsal subiculum.

**Figure 8.**
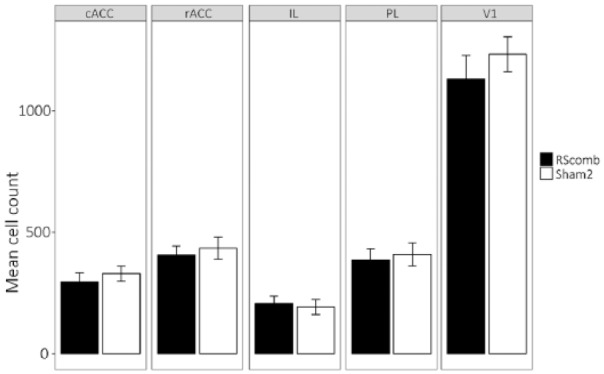
Mean Fos-positive cell counts for cortical regions after combined granular and dysgranular retrosplenial cortex (RSCcomb) and sham control (Sham2) surgeries (Cohort 2). cACC: caudal anterior cingulate; rACC: rostral anterior cingulate; IL: infralimbic cortex; PL: prelimbic cortex; V1: visual cortex.

In the remaining hippocampal and parahippocampal regions (dorsal subiculum, medial entorhinal cortex and post subiculum), there was no main effect of Group (*F* < 1) and no Group × ROI interaction (*F* < 1; Figure 7(a)). Similarly, there was no difference between the Fos-positive cell counts for the two groups in the anteromedial thalamic nucleus (*t* < 1; Figure 7(b)). The cell counts from the cortical regions (prelimbic, infralimbic, anterior cingulate and visual cortices) also showed no main effect of Group (*F* < 1) and no Group × ROI interaction (*F* < 1; Figure 8).

### Subgroup analyses (c-Fos)

As previously explained, the tissue from Cohort 2 was stained in 13 different batches and the resulting cell counts compared with one another. To confirm the validity of this methodology, the mean total counts from each batch were compared. The resulting one-way ANOVA indicated that there was no systematic difference across the repeated staining procedures (*F* < 1).

In addition, five of the RSCcomb group had appreciable amounts of granular retrosplenial cortex sparing in its superficial layers. For this reason, additional statistical analyses involved just those cases (*n* = 8) with the most extensive retrosplenial lesions, that is, excluding the five cases and their sham controls. These further analyses gave almost exactly the same pattern of results as already described. The only difference was that the Group × ROI interaction seen in the septal hippocampal DG no longer reached significance (*F*(1, 14) = 2.6, *p* = 0.13; *ges* *=* 0.05).

#### Effects of combined retrosplenial cortex lesions on distal Zif268 expression (groups RSCcomb)

**Figure 9.**
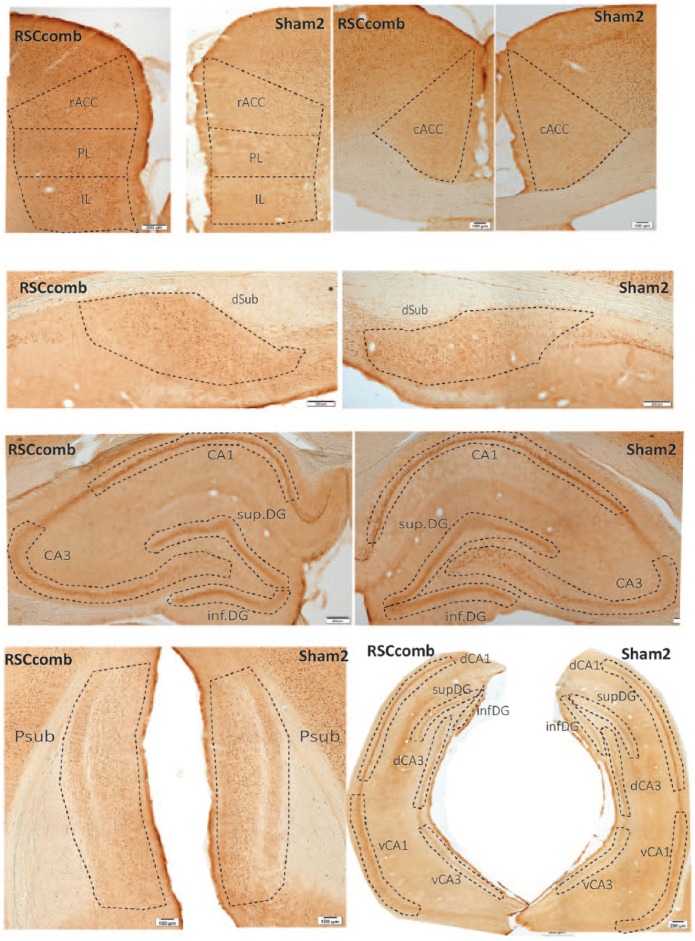
Coronal images of sections stained for *zif268* protein from Cohort 2, showing tissue from the combined granular and dysgranular retrosplenial lesion (RSCcomb) and sham control (Sham2) cases. cACC: caudal anterior cingulate; rACC: rostral anterior cingulate; AM: anteromedial thalamic nucleus; dCA1: dorsal CA1; vCA1: ventral CA1; dCA3: dorsal CA3; vCA3: ventral CA3; infDG: inferior dentate gyrus; supDG: superficial dentate gyrus; mEnt: medial entorhinal cortex; IL: infralimbic cortex; PL: prelimbic cortex; Psub: post subiculum; dSub: dorsal subiculum; V1: visual cortex.

**Figure 10.**
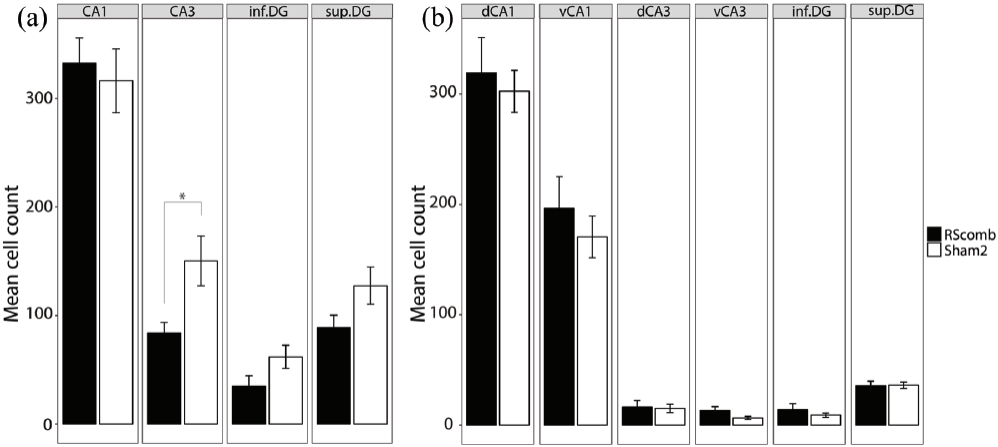
Mean *zif268*-positive cell counts after combined granular and dysgranular retrosplenial cortex (RSCcomb) and sham control (Sham2) surgeries (Cohort 2). (a) Zif counts in septal hippocampal regions. (b) Fos counts in intermediate hippocampal regions. dCA1, dorsal CA1; dCA3, dorsal CA3; vCA1, ventral CA1; vCA3, ventral CA3; inf.DG, inferior dentate gyrus; sup.DG, superficial dentate gyrus. *p ⩽ 0.05.

Figure 9 shows the appearance of Zif268 protein staining in Cohort 2. In the septal hippocampus (Figure 10(a)), there was no main effect of Group (*F*(2, 48) = 2.09, *p* = 0.16; *ges* *=* 0.06), but there was a significant Group × ROI interaction (*F*(2, 48) = 4.48, *p* = 0.02; *ges* *=* 0.06). Simple effects analysis revealed that this interaction was due to higher Zif268-positive cell counts in the Sham2 group relative to the RSCcomb group in CA3 (*F*(1, 24) = 7.15, *p* = 0.01; *ges* *=* 0.23; Figure 10(a)). When the counts in the superior and inferior blades of the DG were analysed separately, there was no significant difference between the two groups, although there was a tendency for lower Zif268-positive cell counts in both sub-regions of the DG in the RSCcomb group relative to the Sham2 group (*F*(1, 24) = 3.78, *p* = 0.06; *ges* *=* 0.12; Figure 10(a)).

In the intermediate hippocampus (Figure 10(b)), there was no main effect of Group and no Group × ROI interaction (*F* < 1). When the Zif268-positive cell counts in the superior and inferior blades of the DG were analysed separately, there was no main effect of Group (*F* < 1) and no Group × ROI interaction (*F*(1, 24) = 1.13, *p* = 0.3; *ges* *=* 0.01; Figure 10(b)). Similarly, when CA1 and CA3 were split into dorsal and ventral subfields, there was no difference in Zif268-positive cell counts between the two groups (*F* < 1; Figure 10(b)).

**Figure 11.**
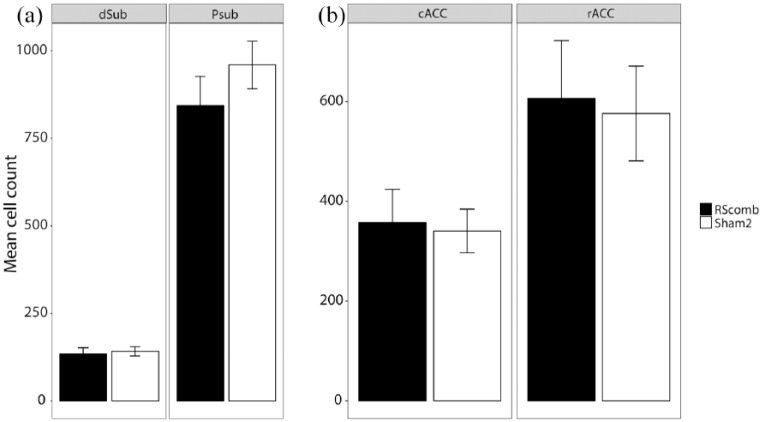
Mean *zif268*-positive cell counts after combined granular and dysgranular retrosplenial cortex (RSCcomb) and sham control (Sham2) surgeries (Cohort 2). (a) Zif counts in the dorsal subiculum (dSub) and post subiculum (Psub). (b) Zif counts in the caudal (cACC) and rostral (rACC) anterior cingulate cortices.

There was no main effect of Group (*F*(1, 24) = 1.05, *p* = 0.32; *ges* *=* 0.03) and no Group × ROI interaction (*F*(1, 24) = 1.26, *p* = 0.27; *ges* *=* 0.02) in the dorsal subiculum and post subiculum (Figure 11). Finally, there was no main effect of Group (*F* < 1) and no Group × ROI interaction (*F* < 1) in the rostral and caudal anterior cingulate cortices (Figure 11).

### Subgroup analyses (Zif268)

As described above, additional statistical analyses involved just those cases (*n* = 8) with the most extensive retrosplenial lesions, that is, excluding the five cases with greater amounts of granular retrosplenial cortex sparing and their sham controls. The main effect of Group remained non-significant (*F* < 1), but now the Group × ROI interaction in septal hippocampus was also non-significant (*F*(2, 28) = 1.78, *p* = 0.19; *ges* *=* 0.04). Similarly, the trend for reduced Zif268-positive cell counts in both sub-regions of the DG in the RSCcomb group relative to the Sham2 group was no longer present (*F*(1, 14) = 1.66, *p* = 0.22; *ges* *=* 0.1). All other comparisons yielded the same pattern of results as for the full group.

## Discussion

Retrosplenial cortex lesions had very limited impact on c-Fos and Zif268 levels across the multiple sites selected, many of which are directly connected with retrosplenial cortex (Sugar et al., 2011; van Groen and Wyss, 1990, 1992, 2003; Wyss and van Groen, 1992). The initial null results for Fos levels in the rats with dysgranular retrosplenial cortex lesions (group RSCdysg, Cohort 1) were essentially replicated and then extended in those rats with more complete retrosplenial surgeries (group RSCcomb, Cohort 2). Of the numerous sites examined in Cohort 2, the only potential Fos reduction was in the septal DG (superior blade). Evidence of lesion-induced hypoactivity was not, however, seen at more caudal (intermediate) hippocampal levels (Cohort 2) nor was it present in the subgroup of RSCcomb cases with the most extensive lesions. For the Zif268 analyses, there was again evidence of reduced activity in the septal hippocampus, but now only in CA3. These Zif268 changes were not, however, seen when the analyses were restricted to those cases with the largest retrosplenial lesions. Once again, no lesion effects were seen in the intermediate hippocampus nor in those other sites examined. The two IEGs, c-*fos* and *zif268*, capture different neuronal responses and so provide complementary perspectives on neuronal activity and change (Frankland and Bontempi, 2005; Guzowski et al., 2001, 2005). This property reinforces the overall picture of marginal, restricted septal hippocampal changes in IEG activity following the loss of retrosplenial cortex tissue, with no apparent changes in the intermediate or temporal hippocampus, nor in the parahippocampal region and related cortices.

Given the restricted pattern of results, it is important to consider the sensitivity of the methods. One possible concern, floor effects in IEG expression, was largely alleviated by allowing the rats to run down pre-selected arms in a radial-arm maze in a novel room. It has repeatedly been shown that in intact animals, there is increased c-*fos* and *zif268* expression in hippocampal, parahippocampal and retrosplenial regions after a variety of radial-arm maze procedures (Jenkins et al., 2002b; Pothuizen et al., 2009; Vann et al., 2000a). Furthermore, the identical behavioural procedure to that in this study was sufficient to show that mammillothalamic tract lesions reduce c-*fos* activity in both the granular and dysgranular retrosplenial cortex, as well as in the septal hippocampus (CA1, CA3 and DG), parasubiculum and post subiculum (Vann and Albasser, 2009; see also, Jenkins et al., 2004; Vann, 2013). Finally, experiments that merely involved placing rats in novel rooms have repeatedly shown how both anterior thalamic and hippocampal lesions reduce c-*fos* expression in retrosplenial cortex (Jenkins et al., 2002b; Vann, 2013; Vann and Albasser, 2009). The clear implication is that it is the lesion site and not the behavioural task (exposure to a novel room) that is responsible for the lack of IEG changes in this study.

Another potential issue concerns power. While the RSCdysg group only contained six rats, the RSCcomb group involved over twice that number of animals, yet still only revealed limited IEG effects, consistent with the reported effect sizes. Furthermore, the RSCcomb group size was greater than in related studies showing reliable c-*fos* and *zif268* activity decreases in multiple limbic areas, for example, following lesions of the mammillothalamic tract, anterior thalamic nuclei and hippocampus (Albasser et al., 2007; Amin et al., 2010; Dalrymple-Alford et al., 2015; Dumont et al., 2012; Frizzati et al., 2016; Jenkins et al., 2002a, 2002b, 2004; Poirier et al., 2011; Poirier and Aggleton, 2009; Vann and Albasser, 2009). Moreover, further corrections of the alpha level, based on the numbers of comparisons (two for Experiment 1 and five for Experiment 2) would mean that none of the main effects is significant. This result underlines the striking paucity of IEG changes after retrosplenial lesions.

It might be supposed that some of the null results could have arisen from incomplete lesions. In practice, the surgeries successfully targeted much of the length of the retrosplenial region, while additional analyses that involved just those RSCcomb cases with the largest lesions consistently failed to reveal IEG activity changes. At the same time, both sets of lesions (RSCdysg and RSCcomb) were sufficient to have demonstrable effects on behavioural tests of learning and memory (Hindley et al., 2014a, 2014b; Powell et al., 2017a, 2017b). For example, the RSCdysg group was impaired on a spatial discrimination task finished 1 month before this study (Hindley et al., 2014b). It might also be supposed that the relatively long interval post surgery (9 months or more) might have provided time for a recovery in IEG expression. At present, different survival times following retrosplenial lesions have not been examined, though studies into the impact of lesions in closely related sites suggest that post-surgical survival time is not the reason for the null effects. Taking the example of anterior thalamic lesions, their distal IEG hypoactivity effects within retrosplenial cortex seem permanent, with consistent evidence that they do not diminish even many months post surgery (Jenkins et al., 2004; Loukavenko et al., 2016; Poirier and Aggleton, 2009). Indeed, the extent of c-*fos* hypoactivity within retrosplenial cortex was found to be greater a year after anterior thalamic lesions than 4 weeks after the comparable surgery (Poirier and Aggleton, 2009). Similarly, studies into the effects of hippocampal and mammillothalamic tract lesions found no evidence of IEG compensation 8 and 9 months post surgery, respectively (Albasser et al., 2007; Vann, 2013). Finally, while it is possible that sites not investigated in this study might have shown lesion-induced changes, this study focussed on those areas that directly receive or provide retrosplenial inputs. These areas included key steps in the historic circuit of limbic connections described by Papez (1937), which incorporates retrosplenial cortex (Bubb et al., 2017).

Reflecting these same connections, retrosplenial cortex lesions in rats can affect tests of spatial memory (Cooper and Mizumori, 1999; Harker and Whishaw, 2002; Keene and Bucci, 2009; Miller et al., 2014; Vann and Aggleton, 2002). One potential contributing factor would be if retrosplenial cortex damage disrupts activity in sites such as the hippocampus, the performance will be impaired. It is already known that temporary inactivation of the retrosplenial cortex with tetracaine can affect ‘place field’ stability within the hippocampus (Cooper and Mizumori, 2001). Furthermore, IEG activation is related to the presence of ‘engrams’ in sites such as the hippocampus (Liu et al., 2014; Tonegawa et al., 2015; Xu and Sudhof, 2013). For these reasons, the very meagre effects of retrosplenial lesions on hippocampal IEG activity are striking.

Instead, the present findings may help to explain apparent differences in the severity of anterior thalamic nuclei and retrosplenial cortex lesion effects on tests of spatial memory, despite their dense interconnectivity (Shibata, 1998; van Groen and Wyss, 2003). While anterior thalamic lesions consistently produce severe, persistent deficits on tasks such as T-maze alternation and radial-arm maze foraging, rats with retrosplenial cortex lesions show milder impairments that often recover with testing (Bubb et al., 2017; Neave et al., 1994; Nelson et al., 2015; Vann and Aggleton, 2004). It is, therefore, possible that anterior thalamic lesions are the more disruptive as they cause additional cortical dysfunctions (Garden et al., 2009) which, as measured by IEG levels, are evident in both retrosplenial cortex and the hippocampus (Dalrymple-Alford et al., 2015; Dumont et al., 2012; Jenkins et al., 2002a, 2002b). This difference is of note as both the retrosplenial cortex and the hippocampus are associated with engram representations of spatial context that depend on c-*fos* activity (Cowansage et al., 2014; Milczarek et al., 2018; Tonegawa et al., 2015). These same issues raise the question of why retrosplenial cortex damage might not have the same deleterious IEG effects as anterior thalamic lesions. One potential explanation concerns the various parallel pathways within their shared network, many of which bypass retrosplenial cortex (Bubb et al., 2017). The situation is quite different for the anterior thalamic nuclei as they are the sole recipients of mammillary body inputs within this network (Vann, 2013) as well as an integrator of hippocampal projections to the diencephalon (Tsanov et al., 2011).

One reason for examining retrosplenial cortex arises from its vital role in supporting human episodic memory (Maguire, 2001; Valenstein et al., 1987; Vann et al., 2009). Interestingly, an amnesic patient with retrosplenial damage was found to show reduced glucose activity in both frontal and anterior thalamic areas (Heilman et al., 1990). While no evidence of comparable changes was found in this study, important differences include the likely involvement of the cingulum bundle in the patient study. Further reasons for studying retrosplenial cortex include its potential contribution to prodromal Alzheimer’s disease (Nestor et al., 2003a, 2003b). An association between the retrosplenial cortex and Alzheimer’s disease is also seen in a transgenic mouse model (Tg2576) that excessively produces β-amyloid (Hsiao et al., 1996). These mice show changes in cytochrome oxidase and c-*fos* activity in retrosplenial cortex, months before detectable plaque deposition (Poirier et al., 2011). Another mouse model (TgaPParc) shows β-amyloid deposition in retrosplenial cortex shortly after its initial appearance in the subiculum (Ronnback et al., 2012), again highlighting the potential significance of this cortical area. While not wishing to suggest that cytotoxic retrosplenial lesions can model pathological processes such as plaque deposition or progressive atrophy, the discovery that sites closely connected with retrosplenial cortex display such apparent resilience following its lesioning remains informative. The implication is that the loss of retrosplenial neurons need not be sufficient to induce a cascade of dysfunctions in related areas. Although this remains a preliminary conclusion, the contrast between the impact of anterior thalamic damage (marked and widespread) and retrosplenial cortex damage (highly restricted) on IEG expression in related sites appears clear-cut.
